# Lipo‐Glc‐1,6‐P_2_ : A Bioprecursor Prodrug for Phosphomannomutase‐2 Congenital Disorder of Glycosylation

**DOI:** 10.1002/iub.70101

**Published:** 2026-04-12

**Authors:** Federica Sodano, Maria Monticelli, Bruno Hay Mele, Barbara Rolando, Loretta Lazzarato, Angela De Simone, Vincenza Andrisano, Debora Paris, Maria Grazia Rimoli, Maria Vittoria Cubellis, Giuseppina Andreotti

**Affiliations:** ^1^ Department of Pharmacy University of Napoli “Federico II” Naples Italy; ^2^ Institute of Biomolecular Chemistry, National Research Council of Italy Pozzuoli Italy; ^3^ Department of Biology University of Napoli “Federico II” Naples Italy; ^4^ Department for the Promotion of Human Science and Quality of Life San Raffaele Open University Rome Italy; ^5^ Department of Drug Science and Technology University of Turin Turin Italy; ^6^ Department for Life Quality Studies University of Bologna Rimini Italy

**Keywords:** glucose‐1,6‐bisphosphate, pharmacological chaperone, PMM2‐CDG, prodrug approach, protein–ligand binding

## Abstract

Phosphomannomutase‐2 (PMM2) deficiency leads to the prominent Congenital Disorder of Glycosylation (CDG), a rare disease currently lacking effective treatment options. The complete absence of PMM2 activity is incompatible with life, and all patients carry at least one missense destabilising variant that allows residual enzymatic function. This makes PMM2‐CDG amenable to pharmacological chaperone treatment. Glucose‐1,6‐bisphosphate (Glc‐1,6‐P_2_) is PMM2's natural activator and stabiliser, but its clinical application is severely limited due to its unfavourable physicochemical profile. Here, we applied the bioprecursor prodrug strategy to design and synthesise Lipo‐Glc‐1,6‐P_2_, a novel prodrug with good stability and oral bioavailability. Its advantageous physicochemical profile was confirmed through metabolomics‐based studies in fibroblasts derived from PMM2‐CDG patient.

**Abbreviations**
CDG: congenital disorders of glycosylation
DMSO: dimethylsulfoxide
Glc‐1,6‐P_2_
: glucose‐1,6‐bisphosphate
Lipo‐Glc‐1,6‐P_2_
: lipophilic glucose‐1,6‐bisphosphate
Man‐1‐P: mannose‐1‐phosphate
Man‐6‐P: mannose‐6‐phosphate
PAMPA: parallel artificial membrane permeability assay
PBS: phosphate‐buffered saline
PCT: pharmacological chaperone therapy
PMM1: phosphomannomutase‐1
PMM2: phosphomannomutase‐2
TCA: tricarboxylic acid

## 1 Introduction

Congenital disorders of glycosylation (CDG) are a heterogeneous group of rare metabolic diseases caused by defects in protein and lipid glycosylation. The condition now known as PMM2‐CDG was first recognised clinically in the 1980s by Jaeken and colleagues, who described a multisystem syndrome with endocrine and neurologic involvement [1]; however, the causal gene was identified only in the late 1990s. In 1997, Matthijs et al. demonstrated that biallelic pathogenic variants in *PMM2*—encoding phosphomannomutase‐2—cause the disorder historically termed CDG‐Ia, establishing PMM2 deficiency as its molecular basis [2]. The scientific community now refers to it as PMM2‐CDG—the most prevalent congenital disorder of glycosylation—affecting over 1000 individuals worldwide [3]. Clinically, PMM2‐CDG presents as a multisystem disorder with prominent central nervous system involvement; natural‐history work and recent reviews detail infantile, childhood and later stages with variable severity [4, 5, 6]. Despite significant disease burden, no disease‐modifying therapy is currently approved; management remains supportive, while multiple experimental strategies are in development [7, 8, 9].

PMM2 plays a crucial role in protein *N*‐glycosylation, catalysing the isomerisation of mannose‐6‐phosphate (Man‐6‐P) to mannose‐1‐phosphate (Man‐1‐P), a precursor of GDP‐mannose. It shares more than 60% sequence identity with its paralog, PMM1 [10]. Both enzymes require glucose‐1,6‐bisphosphate (Glc‐1,6‐P_2_) as an activator for their mutase activities [11, 12, 13]. Additionally, PMM1 exhibits phosphatase activity—including the hydrolysis of Glc‐1,6‐P_2_—which may represent its main physiological role [14].

To date, more than 140 pathological pathogenic variants have been identified in PMM2‐CDG [15]. Most patients are compound heterozygotes, carrying either one inactivating and one hypomorphic variant, or two different hypomorphic variants in heterozygosity; homozygosity for a single hypomorphic variant is rare, while homozygosity for inactivating variants has never been observed, as complete absence of PMM2 activity is incompatible with life, consistent with embryonic lethality seen in *Pmm2*‐null mice [16, 17, 18]. Hypomorphic variants typically impair protein stability. Compound heterozygosity is compatible with the formation of mixed dimers, composed of different PMM2 variants in vivo. Since pathogenic variants generally affect protein stability, pharmacological chaperone therapy (PCT), based on the stabilisation of protein native conformation fold by small molecules [19, 20], represents a rational therapeutic approach for PMM2‐CDG.

Glc‐1,6‐P_2_ has been shown to act as a natural PC for PMM2, by binding and stabilising indeed the enzyme. This effect has been extensively studied in vitro using purified mutant proteins [12, 13]. However, compound heterodimers formed in patients may not respond identically to homodimers, potentially complicating extrapolation from simplified models.

A recent study showed that increasing intracellular Glc‐1,6‐P_2_—via *PMM1* knockout and consequent depletion of its bisphosphatase activity—led to improved glycosylation in PMM2‐CDG fibroblasts [21]. While these results underscore the therapeutic potential of Glc‐1,6‐P_2_, its direct application is limited by poor membrane permeability due to its high polarity.

In this work we designed, synthesised and characterised a membrane‐permeable derivative of Glc‐1,6‐P_2_, named Lipo‐Glc‐1,6‐P_2_. This approach is further supported by previous results in which hydrophobic derivatives of Man‐1‐P were successfully synthesised and shown to elicit significant biological effects [22]. To improve the physicochemical properties of Glc‐1,6‐P_2_, we employed a bioprecursor prodrug strategy [23]. This approach involves the chemical modification of a parent drug through the attachment of enzymatically or chemically cleavable groups to improve membrane permeability and overall pharmacokinetics [24, 25, 26, 27, 28]. Acetyl groups, commonly used for carbohydrate prodrugs, offer favourable properties including ease of introduction and removal and high overall yields. Its advantageous physicochemical profile was further supported by metabolomics‐based studies conducted in fibroblasts derived from PMM2‐CDG patients, strengthening the rationale for its potential therapeutic application.

## 2 Materials and Methods

### 2.1 Cell Cultures

^p.Arg141His/Phe119Leu^PMM2, ^p.Phe119Leu/Phe119Leu^PMM2 and ^p.Arg141His/Val129Met^PMM2 fibroblasts, a gift from Prof. Flemming Skovby (rigshospitalet.dk—informed consent obtained from patients in accordance with The Code of Ethics of the World Medical Association [Declaration of Helsinki]) [17] or obtained from the Telethon Network of Genetic Biobanks, were grown in RPMI medium supplemented with 10% fetal bovine serum, 2 mM glutamine, 0.5 mg/mL penicillin, 0.5 mg/mL streptomycin and non‐essential amino acids at 37°C in 5% humidified CO_2_. Fibroblasts were immortalised following the protocol described in [18]; briefly, cells were co‐infected with HPV16 E6/E7 and hTERT lentiviral vectors (infection number 1). After a week, cells were split and infected again only with hTERT (infection number 2) and cultured until stabilisation.

Table A1 reports the available genetic and demographic information for the cell lines.

### 2.2 Cellular Thermal Shift Assay (CeTSA)

Five millions cells were collected and lysed in 1.4 mL PBS added with protease inhibitors (Roche) through five freeze/thaw cycles, followed by centrifuging at 10,000*g* for 10 min. Protein extracts were incubated in the presence or the absence of 0.5 mM Glc‐1,6‐P_2_ at increasing temperatures (range 25°C–75°C) for 4 min, then centrifuged at 17,000*g* for 20 min. Soluble fractions were denatured, loaded on 15% polyacrylamide gels and run at 200 V for 60 min. Proteins were blotted on PVDF membranes using the Trans‐Blot Turbo Mini PVDF Transfer system, running at 1.3 A (up to 25 V) for 7 min. Membranes were blocked in 5% milk for 30 min at 4°C, washed with Tween 0.05% TBS for 8 min at 4°C, incubated with anti‐PMM2 primary antibody (10666‐1‐AP by Proteintech, 1:500 dilution, overnight incubation at 4°C), washed again and incubated with secondary antibodies for 90 min at 4°C. Upon washing and Tween removal, images were acquired using a ChemiDoc MP Imaging System—PMM2 bands' quantification and analysis allowed the determination of the soluble fraction. Signal intensities were plotted against temperature for each genotype.

### 2.3 Synthesis

All reactions involving air‐sensitive reagents were performed under nitrogen in oven‐dried glassware using the syringe‐septum cap technique. All solvents were purified and degassed before use. Chromatographic separations were carried out under pressure on Merck silica gel 60 using flash‐column techniques. Reactions were monitored by thin‐layer chromatography (TLC), performed on 0.25 mm silica gel‐coated aluminium plates (Merck 60 F254) with visualisation achieved by UV light (254 nm) or ninhydrin staining. Unless otherwise specified, all reagents were used as received without further purification. Dichloromethane (DCM) was dried over P_2_O_5_ and freshly distilled under nitrogen prior to use. *N*,*N*‐dimethylformamide (DMF) was stored over 3 Å molecular sieves. Intermediates and final products were characterised by ^1^H NMR and ^13^C NMR at room temperature using a JEOL ECZ‐R 600, operating at 600 and 150 MHz, respectively, using SiMe_4_ as the internal reference. Chemical shifts (*δ*) are given in parts per million (ppm) and the coupling constants (J) in Hertz (Hz). The following abbreviations were used to designate the multiplicities: s = singlet, d = doublet, t = triplet, m = multiplet. ESI spectra were recorded on a Micromass Quattro API micro (Waters Corporation, Mildford, MA, USA), mass spectrometer. Data were processed using a MassLynx System (Waters).

*2,3,4‐Tri‐O‐acetyl‐α,β‐glucose‐1,6‐bis*(*diphenylphosphonate*) (**2**, Lipo‐Glc‐1,6‐P_2_): To a solution of 2,3,4‐tri‐*O*‐acetyl‐α,*β*‐glucose‐6‐diphenylphosphonate (**1**, *α*/*β* = 70/30, 900 mg, 1.67 mmol) in dry DCM (20 mL) under a positive pressure of nitrogen, was added 4‐dimethylaminopyridine (DMAP, 456 mg, 3.67 mmol) in one portion, followed by dropwise addition of diphenyl phosphoryl chloride (3.67 mmol, 0.8 mL) in dry DCM. The resulting solution was stirred at room temperature for 1 h, then quenched with water (20 mL). The aqueous phase was extracted with DCM (2 × 20 mL). The combined organic layers were washed with brine, dried over Na_2_SO_4_, filtered and concentrated under reduced pressure. The crude product was purified by flash chromatography (eluent: DCM/Acetone, 95/5, v/v) yielding compound **2** as a conformer mixture (*α*/*β* = 90/10), isolated as an oil. Yield: 1.44 g (50%). ^1^H‐NMR (600 MHz, CDCl_3_) *δ* = 1.82 (s, 3H); 2.00 (s, 3H); 2.03 (s, 3H); 4.04–4.06 (m, 0.9H, α‐isomer); 4.10–4.12 (m, 0.1H, β‐isomer); 4.14–4.16 (m, 1.8H, α‐isomer); 4.22–4.26 (m, 0.1H, β‐isomer); 4.32–4.35 (m, 0.1H, β‐isomer); 4.65–4.99 (m 0.1H, β‐isomer); 4.87–4.89 (m 0.9H, α‐isomer); 5.07 (t, *J* = 12 Hz, 0.1H, β‐isomer); 5.13 (t, *J* = 12 Hz, 0.9H, α‐isomer); 5.50 (t, *J* = 12 Hz, 0.9H, α‐isomer); 5.53 (t, *J* = 12 Hz, 0.1H, β‐isomer); 5.97–5.99 (m, 0.9H, α‐isomer); 6.31–6.33 (m, 0.1H, β‐isomer); 7.16–7.28 (m, 12H); 7.33–7.37 (m, 8H). ^13^C‐NMR (150 MHz, CDCl_3_) *δ* = 20.2; 20.4; 20.6; 65.7 (d, *J* = 4.5 Hz); 67.2; 69.1; 69.4; 69.9; 94.7 (d, *J* = 6 Hz); 119.9; 120.0 (d, *J* = 4.5 Hz); 120.1 (d, *J* = 4.5 Hz); 120.3 (d, *J* = 4.5 Hz); 125.6 (d, *J* = 36 Hz); 129.8; 129.9 (d, *J* = 10.5 Hz); 150.1 (d, *J* = 7.5 Hz); 150.2 (d, *J* = 7.5 Hz); 150.3 (d, *J* = 6 Hz); 169.0; 169.7; 170.0. ESI‐MS [M + Na]^+^: *m*/*z* 793.9.

### 2.4 Intracellular Glc‐1,6‐P_2_ Quantification

HEK‐293 cells were treated for 18 h with 80 μM Lipo‐Glc‐1,6‐P_2_, previously dissolved in DMSO. The 10 mM stock solution at, was then diluted in DMEM supplemented with 10% fetal bovine serum, 2 mM glutamine, 0.5 mg/mL penicillin, 0.5 mg/mL streptomycin and 1 mM sodium pyruvate. The final percentage of DMSO did not exceed the value of 0.8% to ensure cells viability. After a 1‐h wash‐out with fresh medium, cells were washed with cold PBS and harvested in 5% perchloric acid; following 15 min 10,000*g* centrifuge at 4°C, metabolites containing supernatant was neutralised with 400 mM K_2_CO_3_ and centrifuged again to remove the insoluble salt, then stored at −80°C until analysis; proteins containing pellet was dissolved in 200 mM NaOH and used for protein quantification Glc‐1,6‐P_2_ levels in the clear extract were analysed using an UHPLC‐MS system (Acquity Ultra Performance LC, Waters Corporation Milford MA, USA) equipped with BSM, SM, CM and PDA detectors. The analytical column was a Phenomenex Synergi 4u POLAR‐RP 80 Å (150 × 4.6 mm × 4 μm particle size). The mobile phase consisted of CH_3_CN/H_2_O containing 3.5 mM Et_3_N and 1.3 mM HCOOH (pH = 5.5) in a 5/95 (v/v) ratio. The flow rate was set to 0.35 mL/min. The column effluent was monitored using a Micromass Quattro micro API mass spectrometer operating in multi‐mode ionisation (ESCI) mode. Quantitative measurements were based on the [M–H]^−^ molecular ion. The MS conditions were as follows: nitrogen drying gas heated to 350°C at a flow rate of 800 L/h; nebulising gas (nitrogen) at 80 L/h; capillary voltage in negative mode set to 3000 V; fragmentor voltage at 30 V. Quantitation of Glc‐1,6‐P_2_ was performed using a calibration curve generated from standard solutions of the compound. Linearity was confirmed over a concentration range of 10–400 μM (*r* ^2^ > 0.996). Results were normalised to total protein content, measured by Bradford assay [29].

### 2.5 Stability in PBS, 5% Perchloric Acid and in Human Serum

A 10 mM stock solution of Lipo‐Glc‐1,6‐P_2_ in DMSO was added to phosphate‐buffered saline (PBS, pH 7.4, 50 mM), preheated to 37°C, or 5% perchloric acid, at 4°C, to achieve a final concentration of 100 μM (1% DMSO). At predetermined time points, aliquots of the reaction mixtures were analysed by RP‐HPLC as described below. The experiment was performed in triplicate. For human serum stability assessment, the 10 mM DMSO stock solution of Lipo‐Glc‐1,6‐P_2_ was added to human serum (male AB plasma, USA origin, sterile‐filtered, Sigma‐Aldrich), preheated to 37°C, to obtain a final concentration of 100 μM (1% DMSO). The mixture was incubated at 37°C ± 0.5°C, and at appropriate time intervals, 300 μL aliquots were collected and mixed with 300 μL of CH_3_CN containing 0.1% TFA for protein precipitation. The samples were sonicated, vortexed and then centrifuged for 10 min at 2150*g*. The resulting supernatants were filtered through 0.45 μm PTFE filters (Alltech) and analysed by RP‐HPLC. The experiment was replicated five times.

RP‐HPLC analysis was performed using a HP 1100 chromatograph system (Agilent Technologies, Palo Alto, CA, USA) equipped with a quaternary pump (model G1311A), a membrane degasser (G1379A), a diode‐array detector (DAD) (model G1315B) integrated into the system. Data were processed using HP ChemStation system (Agilent Technologies). The analytical column was AQUASIL C18 (200 × 4.6 mm, 5 μm particle size; Thermo). The mobile phase consisted of 0.1% aqueous TFA (solvent A) and CH_3_CN 0.1% TFA (solvent B), at a 1.0 mL/min flow rate. Elution was performed in gradient mode as follows: initially 10% of solvent B until 6 min, from 10% to 80% of solvent B between 5 and 10 min, 80% of solvent B until 20 min and from 80% to 10% of solvent B between 20 and 25 min. The injection volume was 20 μL (Rheodyne, Cotati, CA). Column effluent was monitored at 210 nm and 260 nm, with reference to a 700 nm wavelength.

Quantitation of Lipo‐Glc‐1,6‐P_2_ was carried out using a calibration curve obtained by analysing standard solutions of the compound. Linearity was confirmed in the concentration range of 10–200 μM (*r* ^2^ > 0.99). Pseudo‐first order half‐times (*t*_1/2_) for chemical and enzymatic hydrolysis were calculated using an exponential decay model with Graph‐Pad Prism v. 5.0 (GraphPad Software Inc., San Diego, CA).

### 2.6 PAMPA Assay

Quality control compounds with known intestinal permeability—atenolol, carbamazepine, coumarin, norfloxacin, ranitidine hydrochloride (all from Sigma‐Aldrich)—were used to validate the analytic setup. Stock solutions for each reference drug were prepared by dissolving 1 mg of the solid in DMSO (Sigma‐Aldrich) to reach a final concentration of 10 mM. These stock solutions were then diluted in PBS (pH 7.4) containing 0.1% Tween to obtain a final concentration of 25 μM. The DMSO concentration did not exceed 5% of the total volume. The acceptor 96‐well microplate (Multiscreen, MASSACCEPTOR from Millipore) was filled with 180 μL of PBS (pH 7.4) containing 5% DMSO and 0.1% Tween‐80. The donor 96‐well plate (Multiscreen IP Sterile Plate PDVF membrane, pore size is 0.45 μm, MAIPN4510 from Millipore) was pre‐coated with 5 μL of a freshly prepared solution of L‐α‐phosphatidylcholine from egg yolk (Sigma‐Aldrich) in 20 mg/mL dodecane (Sigma‐Aldrich) and incubated at 70°C for 5 min. After lipid coating, the donor wells were filled with 180 μL of either the reference compound solutions or the test compound (Lipo‐Glc‐1,6‐P_2_) at a final concentration of 25 μM. The assembled donor‐acceptor plate ‘sandwich’ was incubated under constant slight shaking (50 rpm) at 30°C for 16 h. During this time, the compounds diffused across the artificial lipid membrane from the donor plate into the acceptor plate, mimicking passive permeability. Data are provided as File S1.

### 2.7 Metabolomics

p.Phe119Leu/Arg141His patient‐derived immortalised fibroblasts were treated for 24 h with 50 μM Lipo‐Glc‐1,6‐P_2_, then washed with PBS and enzymatically detached using trypsin, pelleted in PBS and washed again. Metabolites extraction was performed using methanol:chloroform:water 1:1:1 protocol, according to published protocols [30]. Polar fractions were transferred into glass vials and the solvents were removed under a nitrogen stream at room temperature and stored at −80°C until they were analysed. For NMR analysis, polar fractions were resuspended in phosphate buffer saline (PBS, pH 7.4), containing 10% ^2^H_2_O (to provide a field frequency lock) and 1 mM sodium 3‐trimethylsylyl [2,2,3,3‐2H4] propionate as a chemical shift reference for ^1^H spectra. NMR spectra were acquired on a Bruker Avance III–600 MHz spectrometer (BrukerBioSpin GmbH, Rheinstetten, Germany), equipped with a TCI CryoProbe fitted with a gradient along the *Z*‐axis, at a probe temperature of 27°C. In particular, standard 1D proton spectra and 2D experiments (clean total‐correlation spectroscopy TOCSY and nuclear single quantum coherence HSQC) were acquired providing monodimensional metabolic profiles and homonuclear and nuclear spectra for metabolite identification. Metabolite assignments were achieved by comparing signal chemical shifts with literature [31] and online databases [32]. All 1D spectra were processed and automatically data reduced in bins and arranged as a data matrix with AMIX 3.9.7 package (Bruker Biospin GmbH, Rheinstetten, Germany), then imported into the SIMCA14 package (Umetrics, Umeå, Sweden) for multivariate data analysis.

For all the identified metabolites, *p* values were computed using a moderated *t*‐test, and multiple testing was controlled with the Benjamini–Hochberg false discovery rate (FDR) procedure. Pathway analysis on altered metabolites was performed using Metaboanalyst 5.0 [33] choosing the SMPDB library as a reference.

A small helper R script was used to retrieve KEGG pathways associated with metabolites significantly altered by Lipo‐Glc‐1,6‐P_2_, excluding ubiquitous ‘currency’ compounds (e.g., ATP, NAD^+^). We then focused on the two PMM2‐related pathways—Fructose and mannose metabolism (hsa00051) and amino sugar and nucleotide sugar metabolism (hsa00520)—expanded to first‐degree connected pathways, and overlaid the altered metabolites associated to those pathways onto the two KEGG maps.

Metabolomics data are provided as File S2.

### 2.8 Cell Viability Assay

HEK‐293 cells were grown in DMEM supplemented with 10% Fetal Bovine Serum, 2 mM glutamine, 0.5 mg/mL penicillin, 0.5 mg/mL streptomycin and pyruvate at 37°C in 5% humidified CO_2_, then treated with 0–300 μM Lipo‐Glc‐1,6‐P_2_ for 18 h. Cell viability was assessed through 3‐(4,5‐dimethylthiazol‐2‐yl)‐2,5‐ diphenyltetrazolium bromide (MTT) assay [34]. Cells were incubated with 0.5 mg/mL MTT for 3 h, then 75% isopropanol was added and additional 30 min incubation was performed. Absorbance was recorded at 620 nm using a GENios‐Pro 96/384 multifunction microplate reader (Tecan, Milan, Italy). Data were normalised to the vehicle control, which was set to 100% MTT‐reducing activity, and treatment effects were reported as the percent inhibition relative to this baseline. 0.1 mM cannabidiolic acid (CBDA) was used as positive control for cell toxicity.

### 2.9 Statistics

Statistical analyses were performed in R (version 4.5) [35]. CeTSA data were fitted using a Boltzmann logistic model with an additional parameter (Δ*T*_m_) to account for ligand‐induced shifts in melting temperature—fit via the nls() function in R. Bootstrapping via nls tools::nlsBoot()10,000 replicates [36]. To assess the association between Lipo‐Glc‐1,6‐P_2_ concentration and cell viability, a non‐parametric Spearman's rank correlation analysis was conducted using the corr.test() function. This approach was selected due to its robustness to non‐normal distributions and small sample sizes. To compare values between the Lipo‐Glc‐1,6‐P_2_ and no ligand conditions, we used the Mann–Whitney U test, chosen to avoid assumptions of normality given the small sample sizes and unknown distribution of the data. A one‐tailed test was used based on the expectation that values in the Lipo‐Glc‐1,6‐P_2_ group would be greater than in the no ligand group. Effect size was estimated using the rank‐biserial correlation, which quantifies the magnitude of the difference between groups in a distribution‐free manner. All analyses were conducted using the rstatix package [37].

## 3 Results and Discussion

In this work, we developed Lipo‐Glc‐1,6‐P_2_ as a membrane‐permeable bioprecursor of Glc‐1,6‐P_2_, rationally designed through a lipophilic prodrug approach to overcome the intrinsic low permeability of the parent compound and to facilitate its intracellular delivery in the context of PMM2‐CDG therapy.

To corroborate the ability of Glc‐1,6‐P_2_ to stabilise PMM2 in a more physiological context, we first evaluated its effect in patient‐derived fibroblast lysates using a CeTSA [38]. Cell lines were selected based on practical availability in the context of PMM2‐CDG and on the expectation that the genotypes would be informative for stabilisation‐based strategies. p.Phe119Leu, a frequent variant globally, occurs both as p.Phe119Leu/Arg141His and in homozygosity, while p.Val129Met is relatively common in Italy and has been described in association with p.Arg141His [13]. Both p.Phe119Leu and p.Val129Met variants retain enzymatic activity but are less stable than wt‐PMM2, a property consistent with responsiveness to pharmacological chaperones [12, 39].

**FIGURE 1:**
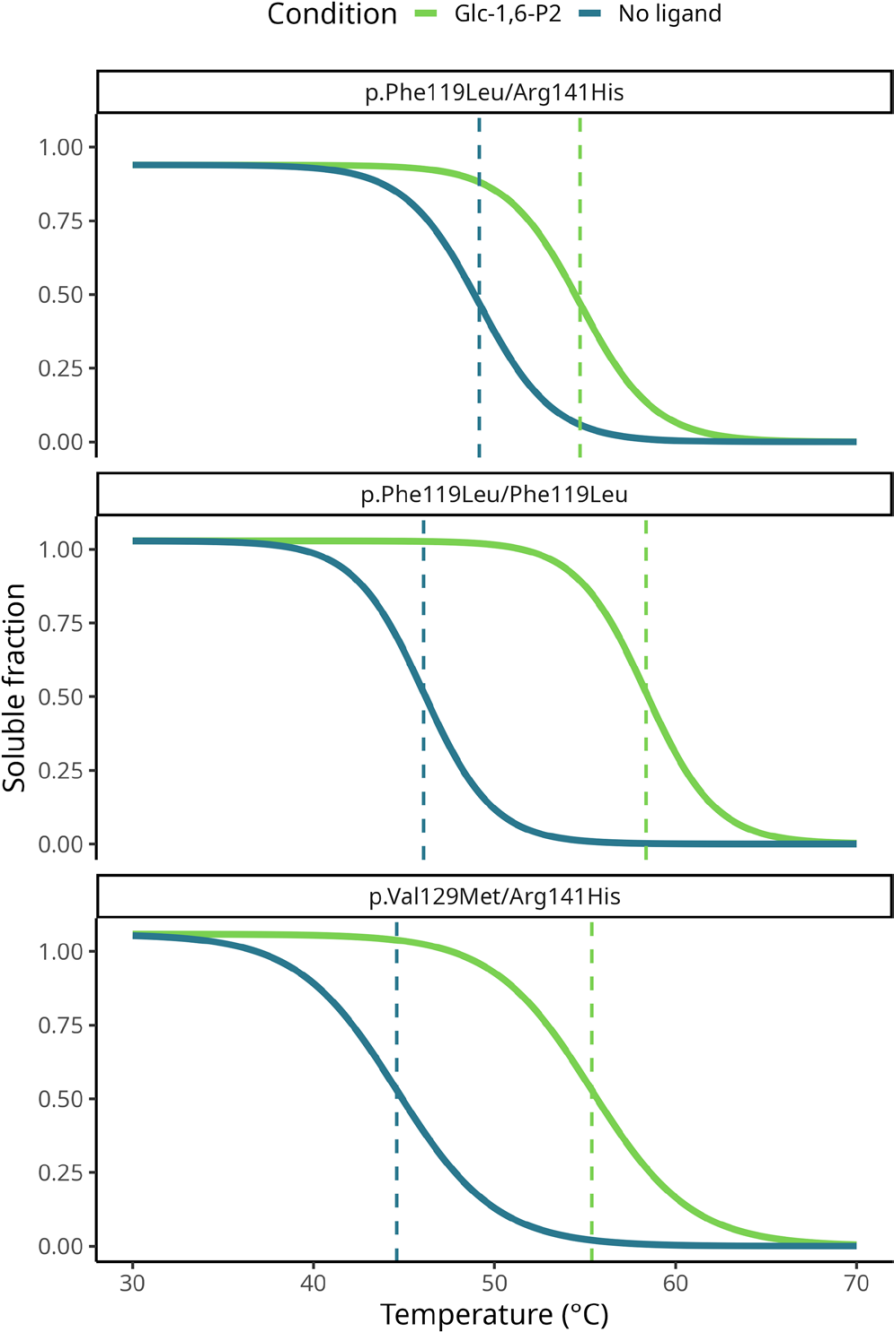
PMM2 melting curves, genotypes shown in facets. Dashed vertical lines indicate the estimated melting Temperature (*T*_m_) for each genotype and condition. Coloured lines distinguish between the experiment carried out in the Glc‐1,6‐P_2_ presence (green line) and in the absence of ligand (blue line). p.Val129Met/Arg141His Δ*T*_m_ = 10.8°C (CI: 8.8°C–12.8°C); p.Phe119Leu/Arg141His Δ*T*_m_ = 5.6°C (CI: 4.6°C–6.5°C); p.Phe119Leu/Phe119Leu Δ*T*_m_ = 12.3°C (CI: 10.5°C–14.0°C).

Our results confirmed that Glc‐1,6‐P_2_ induces thermal stabilisation of PMM2 variants representing common patient genotypes (Figure 1). Specifically, to assess the statistical significance of ligand‐induced stabilisation, 95% confidence intervals (CIs) were estimated for Δ*T*_m_ using bootstrapping. For all genotypes tested, the 95% CI for Δ*T*_m_ excluded zero, supporting a significant stabilising effect of Glc‐1,6‐P_2_.

**SCHEME 1:**
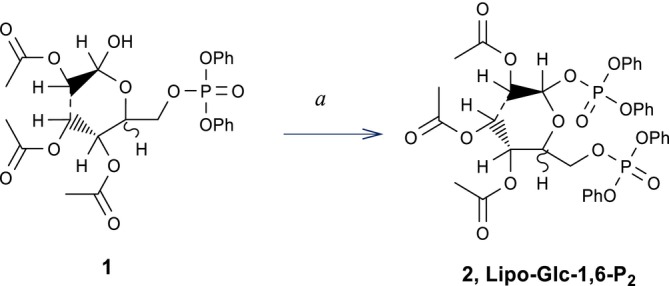
Synthetic procedure of Lipo‐Glc‐1,6‐P_2_. Reaction conditions: (*a*) (PhO)_2_POCl, DMAP, DCM dry, rt, yield 50%.

The prodrug Lipo‐Glc‐1,6‐P_2_ was synthesised following the pathway depicted in Scheme 1. The intermediate triacetyl‐glucose 6‐diphenylphosphate (**1**) was prepared according to established protocols [40, 41]. The final product Lipo‐Glc‐1,6‐P_2_ (**2**) was obtained via esterification of intermediate **1** with diphenyl phosphoryl chloride in the presence of DMAP as a nucleophilic catalyst, using dichloromethane as the solvent, yielding Lipo‐Glc‐1,6‐P_2_ (**2**) as a mixture of α/β conformers.

**FIGURE 2:**
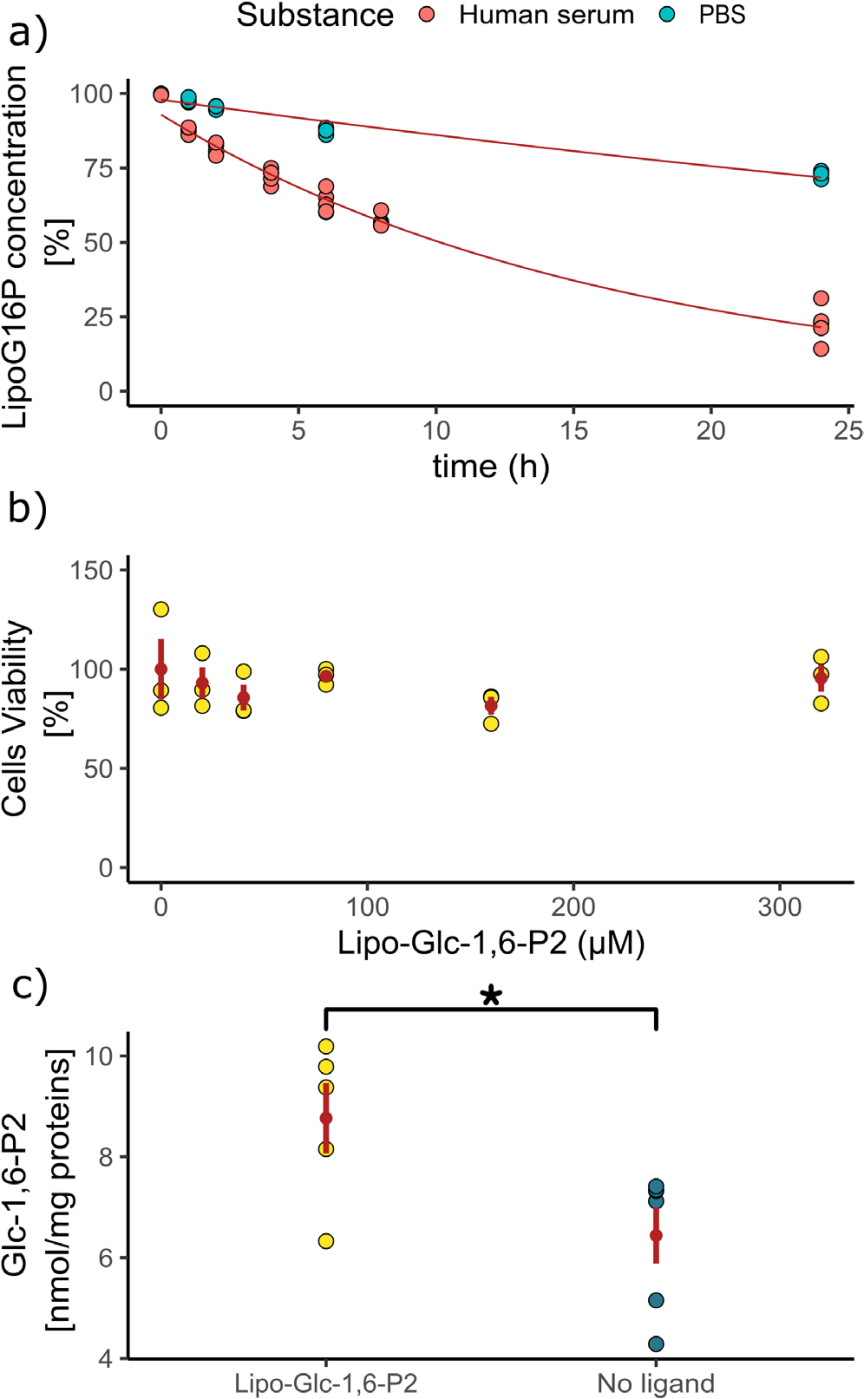
(a) Chemical and enzymatic stability of Lipo‐Glc‐1,6‐P_2_ in PBS and human serum, expressed as % of unmodified compound over 24 h. Calculated half‐time (*t*_1/2_): 10.2 h. (b) Cell viability assay of HEK‐293 cells after 18‐h treatment with Lipo‐Glc‐1,6‐P_2_ (0–330 μM), assessed by MTT assay. Spearman's rank correlation: *ρ* = −0.03, 95% CI [−0.49, 0.45], *p* = 0.912. (c) Intracellular levels of Glc‐1,6‐P_2_ measured by LC‐MS/MS upon treatment with 80 μM Lipo‐Glc‐1,6‐P_2_ for 18 h, followed by 1‐h wash‐out. Mann–Whitney *U* test: *U* = 26, *p* = 0.026; rank‐biserial correlation *r* = 0.61.

The chemical stability of the prodrug was evaluated both in PBS (pH 7.4) at 37°C, and in 5% perchloric acid (HClO_4_) at 4°C, to simulate the physiological conditions and the extraction conditions in the cellular permeability assay, respectively. Lipo‐Glc‐1,6‐P_2_ exhibited relatively high chemical stability: after 24 h incubation in PBS at 37°C, 73% of the initial compound remained; in these conditions its degradation followed first‐order kinetics (Figure 2a). In 5% HClO_4_ at 4°C no degradation was observed (data not shown). Enzymatic stability was assessed in human serum, where the compound underwent more rapid hydrolysis by esterases. After 24 h incubation, 23% of the starting compound remained, with a calculated half‐time (*t*_1/2_) of 10.2 h (Figure 2a). An MTT assay performed on HEK‐293 cells highlighted cell viability upon 18 h treatment up to 320 μM Lipo‐Glc‐1,6‐P_2_ (Figure 2b). Spearman's rank correlation revealed no significant association between Lipo‐Glc‐1,6‐P_2_ concentration increase, and cell viability (%) (*ρ* = −0.03, 95% CI [−0.49, 0.45], *p* = 0.912). This indicates that increasing concentrations of Lipo‐Glc‐1,6‐P_2_ had no detectable monotonic effect on cell viability within the tested range.

PAMPA assay was carried out to assess the oral permeability of Lipo‐Glc‐1,6‐P_2_. Atenolol, carbamazepine, coumarin, norfloxacin and ranitidine hydrochloride, representing drugs with different oral permeabilities, were used as quality controls. The permeability coefficient (*P*_app_), expressed in centimetres per second, was calculated using the following formula [42]:

$$\begin{array}{c} \mathrm{Pe}=\frac{V_{d}\cdot V_{r}}{\left(V_{d}+V_{r}\right)\cdot S\cdot t}\ln\frac{100\cdot V_{d}}{100\cdot \mathrm{Vd}-\%T\left(V_{d}+V_{r}\right)}\\ \%T=\frac{V_{r}\cdot A_{r}}{{\mathrm{Ad}}_{0}\cdot V_{d}}\times 100 \end{array}$$

where *Vd* and *Vr* are the volume of the solutions both in the donor and in the acceptor wells (0.18 cm^3^), *S* is the membrane area (0.266 cm^2^), *t* is the time of incubation expressed in seconds, *Ar* is the absorbance in the acceptor plate after incubation and *Ad*_0_ is the initial absorbance in the donor compartment [43]. A correlation graph for experimental and theoretical *Pe* values of quality control compounds revealed a good correlation between the reported *Pe* [44] and the experimental ones (*R* ^2^ > 0.95). A *Pe* value of 2.76 *×* 10^−6^ cm/s was caluculated for Lipo‐Glc‐1,6‐P_2_, which is indicative of medium oral permeability.

Lipo‐Glc‐1,6‐P_2_ internalisation, and the resulting intracellular increase of Glc‐1,6‐P_2_ were evaluated in HEK293 cells treated with 80 μM of the prodrug. The complete metabolism of the compound was observed after 18 h of incubation followed by a 1‐h wash‐out, as confirmed by LC‐MS/MS analysis. Results are expressed as nmol compound per mg of total proteins. The one‐tailed Mann–Whitney *U* test revealed a statistically significant difference between the Lipo‐Glc‐1,6‐P_2_ and no ligand conditions, *U* = 26, *p* = 0.026, supporting the directional hypothesis. The effect size was large (rank‐biserial correlation *r* = 0.61), indicating a substantial difference in values between the two groups. Thus, the treatment led to a significant increase in intracellular Glc‐1,6‐P_2_ levels (Figure 2c).

**FIGURE 3:**
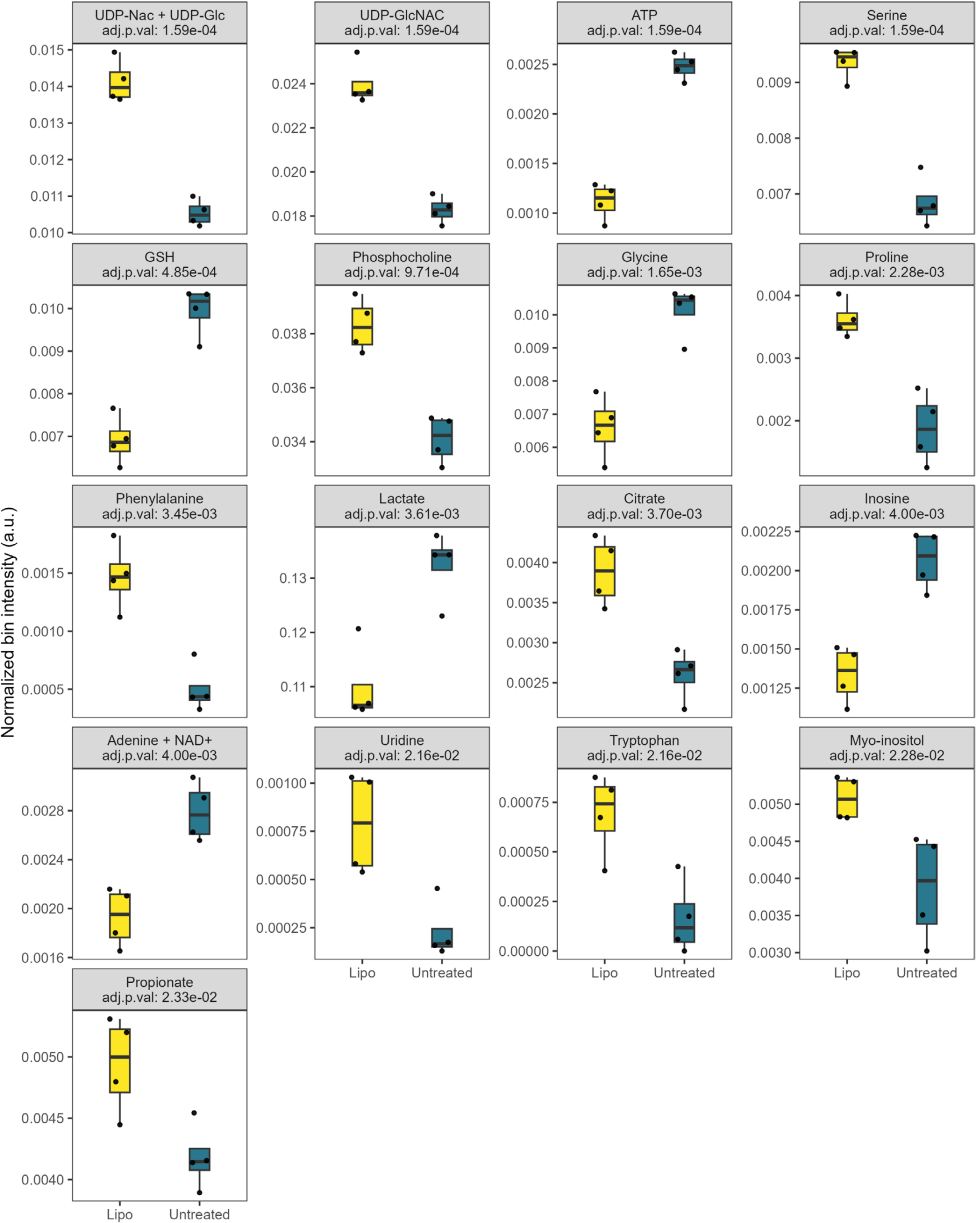
Significantly altered metabolites upon Lipo‐Glc‐1,6‐P_2_ treatment on p.Phe119Leu/Arg141His fibroblasts. Boxplots show normalised bin intensities of metabolites that reached significance in PMM2‐CDG fibroblasts treated with Lipo‐Glc‐1,6‐P_2_ versus controls. Adjusted *p* values (Benjamini–Hochberg FDR) are reported for each metabolite. Significance threshold: FDR < 0.05.

Proton nuclear magnetic resonance spectroscopy (^1^H‐NMR), combined with multivariate statistical analysis, was employed to trace metabolic variations in p.Phe119Leu/Arg141His patient‐derived fibroblasts upon treatment with Lipo‐Glc‐1,6‐P_2_ (Figure 3). A list of all the identified metabolites is provided in File S2. Notably, pathway analysis using the Small Molecule Pathway Database (SMPDB) identified a significant impact on nucleotide sugar metabolism (impact 0.52). These results suggest a recovery of nucleotide‐phosphate pools upon treatment, consistent with previous findings showing increased Glc‐1,6‐P_2_ levels following *PMM1* knock‐out [21]. In parallel, glutathione metabolism and phosphatidylcholine biosynthesis were also affected (impact scores: 0.51 and 0.27, respectively), displaying patterns similar to those observed in *PMM1* knock‐out cells. Intriguingly, inositol and inositol phosphate metabolism were likewise impacted. Since impaired Man‐1‐P production undercuts *N*‐glycosylation, which intersects an inositol‐dependent pathway (GPI) anchor biosynthesis, these changes are mechanistically coherent. More broadly, PMM2‐CDG induces a metabolic profile that mimics cellular high‐glucose stress, characterised by polyol accumulation and disruption of inositol and sugar homeaostasis [45]. Myo‐inositol dysregulation, in particular, is implicated in the secondary pathology of the disease. For example, elevated myo‐inositol levels in the brain have been associated with gliosis in response to neurodegeneration [46]. Interestingly, in our study, treatment with Lipo‐Glc‐1,6‐P_2_ led to a reduction in myo‐inositol levels, aligning with those observed upon *PMM1* knock‐out, suggesting a correction of the altered inositol metabolism.

Non‐currency metabolites proximal to PMM2 and perturbed by Lipo‐Glc‐1,6‐P_2_ treatment are shown in Figure S1.

The protective groups of Lipo‐Glc‐1,6‐P_2_ are expected to be enzymatically cleaved following uptake. Acetyl groups are typically removed by intracellular esterases, releasing acetate, which is readily incorporated into central metabolic pathways [47]. Diphenyl phosphate moieties are likewise hydrolysed, yielding phenol and phosphate. Both by‐products are efficiently processed: acetate enters the tricarboxylic acid (TCA) cycle, while phenol undergoes conjugation (glucuronidation or sulfation) and subsequent excretion [48, 49]. Given the low concentrations employed, these metabolites are unlikely to interfere with key biosynthetic pathways, including those related to glycan or carbohydrate metabolism. Nonetheless, potential effects on acetylation dynamics or energy balance at higher concentrations cannot be excluded and warrant further investigation [50].

Prior in vitro studies have shown that Glc‐1,6‐P_2_ enhances PMM2 activity in a concentration‐dependent manner and promotes the active dimer over the less active/inactive monomer [12, 51]. Notably, the intracellular concentration of Glc‐1,6‐P_2_ is ~80 μM [52], and within this micromolar range, even minor increases can meaningfully impact PMM2 catalytic output. In this framework, Lipo‐Glc‐1,6‐P_2_ is intended to favour catalytically competent PMM2. Thus, an increase in PMM2 quantity in cell extracts after treatment is not expected, and its measurement may underreport the pharmacological effect. Because of this, PMM enzyme activity assays on cell protein extracts were not used to evaluate the prodrug potential of the molecule.

Nevertheless, the current work was primarily focused on chemical synthesis, chemical–physical characterisation and preliminary evidence of a beneficial effect of Lipo‐Glc‐1,6‐P_2_ treatment on PMM2‐CDG fibroblasts. We strongly believe that future directions not addressed in this study include a deeper characterisation of the glycomic effects of Lipo‐Glc‐1,6‐P_2_ and an expansion of the genotype panel. Moreover, administration properties, including dose and exposure timing, will require systematic optimisation, as the efficiency of intracellular conversion of Lipo‐Glc‐1,6‐P_2_ may influence the effective intracellular availability of Glc‐1,6‐P_2_.

## 4 Conclusions

Previous results demonstrated that increasing intracellular levels of Glc‐1,6‐P_2_ can have a beneficial impact on key aspects of the PMM2‐CDG metabolic phenotype, supporting its potential role as a therapeutic target [21]. Building on this evidence, we explored a prodrug‐based chemical modification strategy to enhance the lipophilicity and cellular uptake of Glc‐1,6‐P_2_. This approach proved feasible and lays the foundation for further development of optimised derivatives capable of restoring metabolic balance in PMM2‐deficient cells. These findings highlight the therapeutic promise of Glc‐1,6‐P_2_ supplementation through rational chemical design, offering a novel direction for the treatment of PMM2‐CDG. While our conclusions are supported by previous evidence on Glc‐1,6‐P_2_, the present study primarily demonstrates the feasibility of a prodrug‐based strategy, and does not yet directly address the impact of Lipo‐Glc‐1,6‐P_2_ on PMM2 enzymatic activity and glycosylation.

## Funding

This study was supported by the Italian Ministry of University and Research PRIN 2022B2N2BY (B.H.M., M.V.C.). Accordo per la coesione della Regione Campania—Promozione di progetti di ricerca, sviluppo sperimentale e innovazione collaborativi nel campo delle malattie rare—Intervento no. 28‐CUP: B83C25000740002 (G.A.).

## Conflicts of Interest

The authors declare no conflicts of interest.

## Supporting information


**Figure S1:** Non‐currency metabolites proximal to PMM2 and perturbed by Lipo‐Glc‐1,6‐P_2_ treatment.

**File S1:** PAMPA assay.

**File S2:** Metabolomics data.

## Data Availability

The data that supports the findings of this study are available in the Supporting Information of this article.

## Appendix A See Table A1

**TABLE A1:** Information for the cell lines used in this study.

PMM2	Allele 1	Allele P2	Sex	Age at biopsy	Origin tissue
p.Val129Met/Arg141His	c.385G>A	c.422G>A	M	n.a.	Dermal fibroblasts
p.Phe119Leu/Phe119Leu	c.355T>C	c.355 T>C	M	Paediatric age‐range	Dermal fibroblasts
Phe119Leu/Arg141His	c.355T>C	c.422G>A	M	Paediatric age‐range	Dermal fibroblasts
