# Supplementary material for: Lipo‐Glc‐1,6‐P2 : A Bioprecursor Prodrug for Phosphomannomutase‐2 Congenital Disorder of Glycosylation
Source: IUBMB Life. 2026 Apr 12;78(4):e70101. doi: 10.1002/iub.70101 (PMC13071126; doi:10.1002/iub.70101)
Supplement: Supplementary file 1 — Figure S1: Non‐currency metabolites proximal to PMM2 and perturbed by Lipo‐Glc‐1,6‐P2 treatment. [file IUB-78-0-s003.pdf]

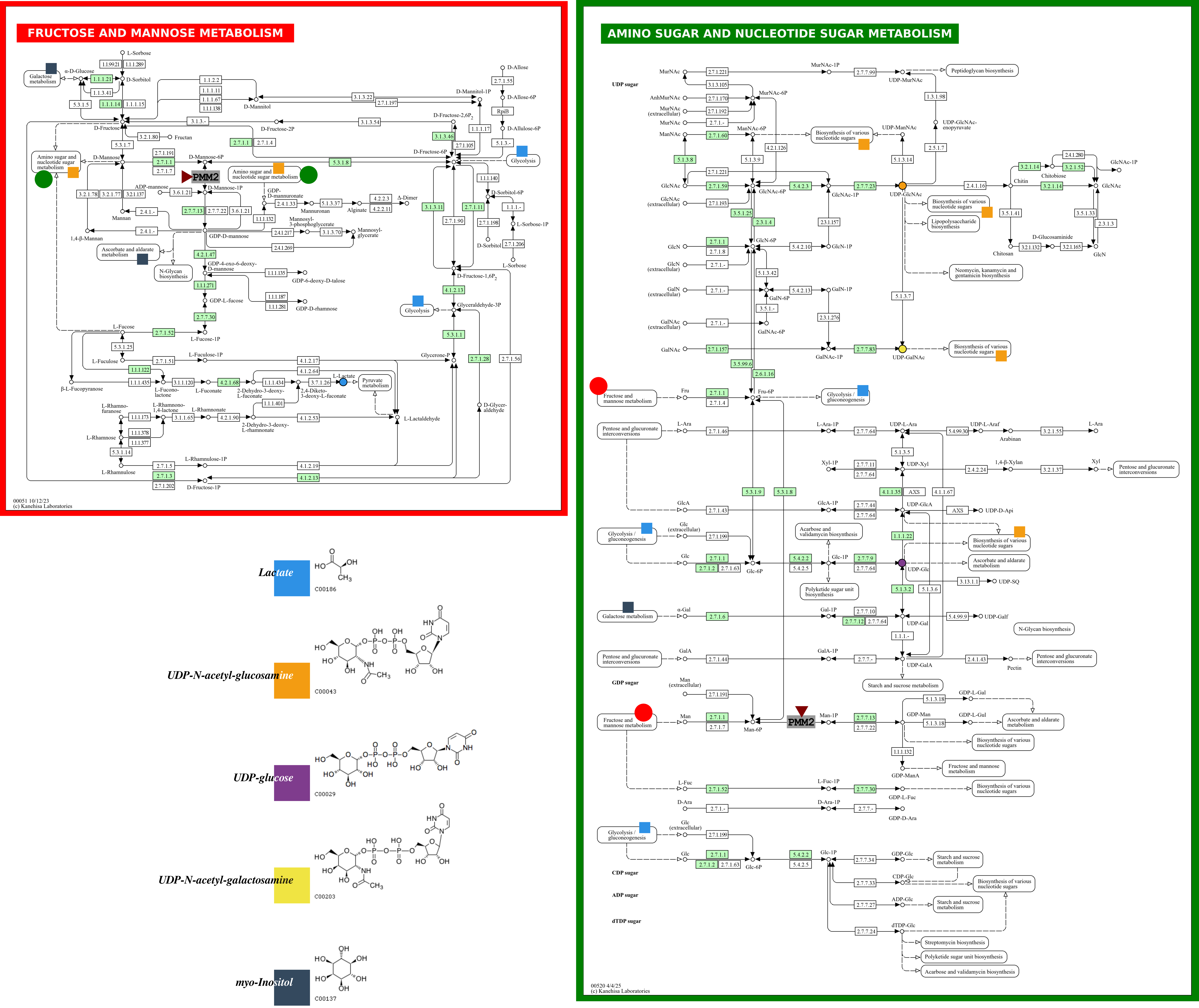

"Supporting Figure 1. Non-currency metabolites proximal to PMM2 and perturbed by Lipo-Glc-1,6-P_2 treatment. KEGG pathway maps for Fructose and mannose metabolism (hsa00051) and Amino sugar and nucleotide sugar metabolism (hsa00520) are shown; and the significantly changed metabolites identified in our dataset and associated to first-degree neighbors of the PMM2 pathways are marked as rectangles (if associated to neighbors) or circles (if within displayed pathway): lactate (C00186) in blue; UDP-GlcNAc (C00043) in orange; UDP-glucose (C00029) in purple; UDP-GalNAc (C00203) in yellow; myo-inositol (C00137) in slate. Each pathway is referenced in the other as filled circle (green for hsa00520 and red for hsa00051) "
